# Gender biases in attributions of blame for workplace mistreatment: a video experiment on the effect of perpetrator and target gender

**DOI:** 10.3389/fpsyg.2023.1161735

**Published:** 2023-06-30

**Authors:** Eva Zedlacher, Takuya Yanagida

**Affiliations:** ^1^Department of Business and Management, Webster Vienna Private University, Vienna, Austria; ^2^Department of Developmental and Educational Psychology, Faculty of Psychology, University of Vienna, Vienna, Austria

**Keywords:** workplace mistreatment, gender bias, attributions of blame, social role theory, perpetrator intent, victim-blaming, video experiments, moral anger

## Abstract

**Introduction:**

Ambiguous psychological workplace mistreatment such as insulting or ignoring a co-worker might trigger gender bias. This study aims to examine whether female perpetrators receive more moral anger and blame from observers than men.

**Methods:**

A sample of Austrian workforce members (*n* = 880, 55.00% women, 44.89% men, 0.11% diverse) responded to standardized videos showing a perpetrator’s angry insult and a perpetrator’s exclusion of a co-worker from lunch. In total, we edited 32 video clips with four female and four male professional actors. We manipulated the following variables: 2 perpetrator gender (male/female) * 2 target gender (male/female) * 2 types of mistreatment (insult/exclusion).

**Results:**

As hypothesized, linear mixed-effects modeling revealed more moral anger and attributions of intent against female perpetrators than against men. Significant three-way interactions showed that female perpetrators were judged more harshly than men when the target was female and the mistreatment was exclusion. Female targets were blamed less when the perpetrator was female rather than male. Male targets did not evoke attributional biases. Observer gender had no significant interaction with perpetrator or target gender.

**Discussion:**

Our findings suggest that gender biases in perpetrator-blaming are dependent on target gender and type of mistreatment. The stereotype of women having it out for other women or being “too sensitive” when mistreated by men requires more attention in organizational anti-bias trainings.

## 1. Introduction

*Workplace mistreatment* is an overarching term for harmful, yet potentially ambiguous social interactions in organizations (Hershcovis et al., 2020). It may involve overt aggression (e.g., screaming, insulting), but also indirect or subtle behaviors (e.g., ignoring, excluding). Assigning men to the role of the “perpetrator” (the agent of the mistreatment) and women to the role of the “target”* (the receiver of mistreatment) fits a common stereotype for deviant workplace behavior (e.g., Weber et al., 2018; Reynolds et al., 2020). This is despite the fact that women just like men engage in workplace mistreatment, and both men and women may become targets of mistreatment (Salin and Hoel, 2013).

In this experimental study our main interest is to find out whether women in the perpetrator role receive more moral anger and dispositional attributions (for instance, a malicious intent) for workplace mistreatment than male perpetrators. An unconscious or implicit gender bias is an observer error of judgment and occurs when a person unconsciously makes gender-distinct evaluations based on gender stereotypes (Madsen and Aradne, 2018). Women mistreating co-workers are likely to violate persistent gender stereotypes and social role expectations that they are (and should be) warm and communal-oriented, while men are (and should be) assertive (Eagly and Wood, 2012; Zedlacher and Salin, 2021).

Gender biases in the workplace mistreatment context have been mainly investigated with written vignettes where male and female names for the respective roles were interchanged (e.g., Brown and Sumner, 2006; Salin, 2011; Sheppard and Aquino, 2013). Results suggest that the gender of the target may also play a role in how a perpetrator’s behavior is evaluated. For example, Salin (2011) found that a severe conflict described in the vignette was least likely to be labeled as “workplace bullying” when the perpetrator was male and the target was female. The construction of the “guilty victim” (Christie, 1986) is a well-researched phenomenon in the context of sexual harassment and assaults against women (see Grubb and Turner, 2012). However, little is known about gender biases in perpetrator- and target-blaming when the target of the workplace mistreatment is male (see Graham, 2006). Moreover, prior studies have not taken into account how the respective vignette description and the type of conflict/mistreatment may have affected gender biases in observer responses. In the current research, gender biases are investigated and discussed for two types of anti-communal mistreatment at work (an angry insult and social exclusion of a target) via standardized video clips with professional actors.

This study contributes to a better understanding of attributions and blame patterns for workplace mistreatment in the following ways: First, attribution theory suggests that people, in particular observers, tend to attribute behavior which disconfirms situational or (gender) role expectations to stereotypical dispositions of the actor rather than the situation (Zedlacher and Salin, 2021; see also Kelley and Michela, 1980; Weiner, 2010). Ambiguity and impoverished information about the actor are considered to facilitate gender bias and cognitive distortion in information processing (Heilman, 2012) and causal attributions. However, so far, most experiments on blame biases stem from severe, often oppressively gendered sexualized or physical crime contexts with clear perpetrator and victim roles. Moreover, experiments often focus on the impact of observer gender regarding victim blaming and perceived deservingness of (female) victims, i.e., with no or little focus on perpetrator gender (for instance, Gerber et al., 2004; Rye et al., 2006; Ayala et al., 2018, for further information see the review by Grubb and Turner, 2012). The present study focuses on attributions of intent and controllability as dispositional and relevant dimensions of blame against perpetrators. We control for the context via comparing two “ambiguous,” yet common types of mistreatment at work, where gendered beliefs and attributional biases toward agents and receivers of the mistreatment may manifest more subtly than in overt (physical) mistreatment.

Second, while the hypotheses of this study focus on the effect of perpetrator gender on specific outcome variables, our study also unveils the relevance of target gender in attributions of blame. Target-blaming is often understood as assigning causal responsibility for the mistreatment to the target, for instance, because the target has provoked the perpetrator (Mulder et al., 2017). In our study, we focus on how observers evaluate a target’s reaction and a target’s coping with the perpetrator’s behavior, to find out whether stereotypes against “sensitive” female targets may reduce the blame against the perpetrator.

Finally, with our innovative, yet standardized video vignettes we aim to answer the call for more contextualization of workplace mistreatment research (Hershcovis et al., 2020) and extend the external validity of extend the external validity of research on attributions of blame. In written vignettes respondents often tend to focus on the target’s behavior and deservingness (Sleed et al., 2002), which might keep them from engaging in more complex and so-called conjunctive blame patterns where blame is distributed among different parties (for instance, Zedlacher and Snowden, 2023). A rich description of the context and the actors involved via electronic vignettes can give detailed insights into (biased) attributions for blame for workplace mistreatment—despite the risk of losing internal validity.

## 2. Theoretical background

### 2.1. Workplace mistreatment and gender

Interpersonal mistreatment at work can take many forms. It can be direct and active (e.g., screaming or shouting), but also indirect (e.g., spreading rumors behind one’s back). Even passive or “non-behaviors” such as withholding information or not inviting a single co-worker to a meeting or social gathering are common examples of mistreatment at work (see Einarsen et al., 2020). A recent meta-analysis by Dhanani et al. (2021) revealed that 34% of workforce members experienced mistreatment and 44% of workforce members witnessed mistreatment at work.

Acts of interpersonal workplace mistreatment may also manifest in various constructs such as *workplace incivility*, *workplace bullying*, or *workplace aggression* (for an overview of criteria and definitional overlaps or differences between the constructs see Hershcovis, 2011). One of the key distinctions between the constructs is the attribution of intent. According to the definition of incivility at work, the intent of the perpetrator to harm the target through uncivil acts (e.g., talking loudly in the office room) is ambiguous (Schilpzand et al., 2016; Cortina et al., 2017). When targets or observers label a behavior “workplace bullying” or “aggression”, they often attribute a malicious intent by the perpetrator in organizational practice (e.g., Saunders et al., 2007; Zedlacher and Salin, 2021). However, it is a challenge to empirically assess the intent of perpetrators. In addition, (direct) observers and targets may differ in their attribution of perpetrator intent (Hershcovis et al., 2007; Einarsen et al., 2020). Apart from methodological challenges, even acts perceived as intentional as well as harmful may be legitimized by non-targets. For example, organizational third parties often excuse screaming or insults with contextual requirements such as work pressure or by attributing (part of the) blame to the target (e.g., Harrington et al., 2015; Mawdsley and Thirlwall, 2019; Zedlacher and Salin, 2021).

As argued in the introduction, observers may expect women in the target role, and men in the perpetrator role due to gender stereotypes and the existing division of labor and roles. Indeed, men more than women are reported as perpetrators of workplace aggression (Hershcovis et al., 2007) and workplace bullying (Salin, 2021). However, unlike in sexual harassment, men are also often reported as targets of workplace aggression and bullying at work. Regarding victimization, women more often self-report as targets of aggression and workplace bullying than men, in particular when in a minority group or leadership position (e.g., Koeszegi et al., 2014; see also Salin, 2021). For the workplace mistreatment context, written vignette studies, where the gender of both protagonists is manipulated, exist. However the labeling and type/content of the mistreatment differ widely across studies (e.g., Brown and Sumner, 2006; Salin, 2011; Sheppard and Aquino, 2013; McCormack et al., 2018). Salin (2021) contends in her review on workplace bullying that other men typically bully men while women are bullied by men and women in more equal proportions. She further proposes that a male perpetrator/female target configuration is the most frequent gender configuration for workplace bullying incidents. McCormack et al. (2018) argue that men bully both men and women, but women rather bully women. Sheppard and Aquino (2017) argue that female/female and male/male combinations are the most common gender configurations for conflicts and harassment in organizations. Dominant power relations in organizations, oppressed group behavior, token dynamics as well as gender segregation at work may to some extent also explain the (mixed) evidence about the prevalence of female/female or male/male dyadic mistreatment configurations (Salin and Hoel, 2013). As women often work in female-dominated domains and at a lower hierarchical level, they often can only mistreat other female co-workers, often via person-related acts and social exclusion rather than work-related acts (Salin and Hoel, 2013).

### 2.2. Moral anger and blame for mistreatment

Harvey et al. (2014) claim that attributions of causes are an integral part of individuals’ cognitive processing of workplace events, and they are affected by the outcomes of the events. If workplace mistreatment disconfirms situational/gender role expectations and requirements, it is likely that observers find the cause of the mistreatment in the perpetrator’s disposition (e.g., a malicious intent to hurt the target; see also Zedlacher and Salin, 2021). However, the primary emotional appraisal third parties make when witnessing another’s (undeserved) mistreatment is moral anger (Mitchell et al., 2015). It is an intense emotional state and deontic response rather than a deliberate cognitive reasoning process (Haidt, 2001; O’Reilly et al., 2016).

In Malle et al. (2014) model of blame, after detection of the moral violation and establishment of agent causality (i.e., that the agent indeed caused the outcome), a cognitive reasoning process involving judgment of intentionality makes up the critical third step. As authors state “*Even if the negativity of the event has already been evaluated, proper blame cannot be assigned to the agent until the question of intentionality has been answered*” (Malle et al., 2014, p. 317). Empirical evidence demonstrates that an observer’s moral anger about interpersonal mistreatment or injustice is closely related to the perception that the perpetrator acted with intent (Russell and Giner-Sorolla, 2011; Umphress et al., 2013). Perceived intent involves criteria such as causation, foreseeability of harm, lack of coercion, and awareness of harm doing (Shaver, 1985; see Zedlacher and Salin, 2021). While perceptions of intent and causal control (lack of impulse or reflex) may overlap substantially, we do not use the terms interchangeably (Yao and Siegel, 2021). For example, small children might be perceived as beating other children with the intent to harm, but they might still be attributed little internal control over the behavior (Malle et al., 2014). However, we propose that both perceived intent and internal control need to be checked off in third-party attributions of perpetrator blame in workplace mistreatment.

Importantly, third parties might often attribute intentionality retrospectively depending on the severity and outcome of the act, i.e., to which extent the target seemed hurt by the act (e.g., Knobe, 2003; O’Reilly et al., 2016; Ng et al., 2020; Zedlacher and Salin, 2021). Targets are not always assigned “victim status” (Christie, 1986). The term “ideal victim” implies that victim status is socially constructed and that guilty or offensive victims exist (Daly, 2014; Christie, 1986; see for instance Rye et al., 2006). Fucci et al. (2021) argue that the ascribed cause or responsibility of the target for his/her suffering is of great importance for third-party attributions of blame as well as for third parties’ intentions to help the target. Perceived *deservingness* is constituted by perceived personality or ascribed behavior of the target that led to the mistreatment (Dhanani and LaPalme, 2019), but also by the target’s coping strategy in response to the mistreatment (“continuation responsibility,” Mulder et al., 2017; see Zedlacher and Snowden, 2023). In this context, Umphress et al. (2013) found across three studies that for observers of workplace mistreatment the perceived intent of the perpetrator to harm the target was more decisive for perceived injustice than the perception of actual harm inflicted on the target.

### 2.3. Hypotheses for gender biases

*Social Role Theory* (Eagly and Wood, 2012) suggests different social expectations for men and women because of a gendered division of labor in society. Communal and relationship-oriented behavior is more frequently observed in women because they occupy jobs that involve care tasks and require empathy, whereas men often occupy positions that require assertiveness and agency. This division of labor leads to the ascription of distinct “traits” to men and women as gender stereotypes (Eagly and Wood, 2012; Zedlacher and Salin, 2021). Gender stereotypes also are prescriptive as they also create normative standards about how men and women should (not) be and should (not) behave. A violation of gender stereotypes might then even trigger instances of severe moral outrage (Heilman, 2012).

There is prior empirical evidence that women face more negative outcomes than men for “anti-communal” behavior. For example, in two field studies by Tai et al. (2022), women experienced more mistreatment when they showed high levels of bottom-line mentality, i.e., when they focused on securing bottom-line outcomes and neglecting competing non-financial organizational goals, while men were punished more for low bottom-line mentality (Tai et al., 2022, see also Greenbaum et al., 2012). Barrett and Bliss-Moreau (2009) found in their experiments that even when respondents received situational information about the causes of emotional expression in target faces, angry faces by women were often traced back to dispositional traits (“She is emotional”). In contrast, men’s angry faces were attributed to unstable and/or situational causes (“He is having a bad day”).

However, prior research from the broader workplace mistreatment context suggests that the impact of perpetrator gender on observer reactions to mistreatment is dependent on the target gender and the respective context and type of “conflict”: Salin (2011) found that the gender configuration of women as targets of men in a conflict scenario was least likely to be labeled “workplace bullying” by male observers in contrast to other gender configurations. In their experimental study, Sheppard and Aquino (2013) found that participant reactions were particularly strong when the “moderate workplace conflict” was described as a conflict between two women. Results might to some extent be related to stereotypes about women having it out for other women (“cat fights”; Sheppard and Aquino, 2013). Existing stereotypes about lack of solidarity and “queen bees” who reject other women since they are a threat to their position (Derks et al., 2011), might affect layperson’s interpretations and the “problematization” of female/female conflicts (Sheppard and Aquino, 2013; Sheppard and Aquino, 2017). In contrast, women as targets of male aggression might face harsher blame for lack of coping, for instance being (too) “emotional” and hence less-credible targets of “assertive” men (Salin, 2011). Men labeled as *victims* of workplace mistreatment may violate the social role expectation of being strong and assertive (Salin, 2003). While even observers might expect women to occupy the target/victim role rather than men (e.g., Reynolds et al., 2020), a male target who is hurt by ambiguous workplace mistreatment might nevertheless face fewer stereotypes about hypersensitivity than a woman. We do not expect significant same-gender differences for male/male configurations as this configuration has not received sufficient attention in the popular discourse.

To sum up, female perpetrators might trigger more moral anger among observers than male perpetrators due to the disconfirmation of communal orientation. Moral anger might be in particular strong when women turn against targets of their gender than against other men. Female targets of female perpetrators might appear more hurt than when the perpetrator is male.

This leads to our first set of hypotheses:

*Hypothesis 1a*: *Moral anger toward perpetrators differs by perpetrator gender: Female perpetrators will receive more moral anger than male perpetrators*.

*Hypothesis 1b*: *There is an interaction between perpetrator and target gender: Female perpetrators of female targets will receive more moral anger than male perpetrators of female targets*.

While moral anger usually comes quickly and without deliberate reasoning, we expect the attribution of intent to involve (longer) sensemaking about causes and possible excuses for the perpetrator’s behavior. Nevertheless, we also expect women to receive more blame than men for both types of workplace mistreatment, in particular when they mistreat other women. This leads to our next set of hypotheses:

*Hypothesis 2a*: *Blame against the perpetrator differs by perpetrator gender: Female perpetrators will receive more blame (intent and control) than male perpetrators*.

*Hypothesis 2b*: *There is an interaction between perpetrator and target gender: Female perpetrators of female targets will receive more blame (intent and control) than male perpetrators of female targets*.

As explained before, target-blaming is a common third-party attribution in workplace mistreatment. Even when perpetrators are blamed, targets might receive a share of the blame (e.g., Zedlacher and Snowden, 2023), in particular regarding the adequate coping strategy upon ambiguous mistreatment (see Mulder et al., 2017). We argue that blaming targets for wrong coping depends on perpetrator gender (Sheppard and Aquino, 2013): Female perpetrators more than male perpetrators might reduce observers’ attributions of blame against both male and female targets. In alignment with the preceding hypotheses on perpetrator-blaming, we further assume that being a female target of a female perpetrator will foster this tendency. This leads to our last set of hypotheses:

*Hypothesis 3a*: *Blame against the target differs by perpetrator gender: Targets of female perpetrators will receive less blame than targets of male perpetrators*.

*Hypothesis 3b*: *There is an interaction between perpetrator and target gender: Female targets of female perpetrators will receive less blame than male targets of female perpetrators*.

The current research studies the effects of perpetrator and target gender on observer reactions via an experimental video study. Since the context and type of mistreatment is likely to affect perceptions, we test whether our results are robust across two common types of mistreatment, angry insult and exclusion.

## 3. Methods

This study has received funding from the Austrian Chamber of Labor (section Lower Austria) as part of a larger project on fighting workplace mistreatment by raising awareness of bystander behavior and intervention in workplace mistreatment.

### 3.1. Participants and procedure

The survey was distributed via the *Qualtrics* survey tool and with the support of a professional research institute in Fall 2021. The research institute recruited members from an established panel of Austrian workforce members who received incentives via points. The sampling strategy was to reach a fairly balanced sample of active Austrian workforce members regarding industry, gender, and age. In the informed consent form, participants were asked to participate in a video study that tackles “difficult situations” between employees at work. Participants were also informed that they were going to watch two episodes with different employees from the same Austrian company that had suffered from distribution problems with suppliers lately.

#### 3.1.1. Information check

We made sure that respondents had watched all episodes entirely by asking questions related to the content of each mistreatment type and/or probing respondents with questions by mixing up (different) names of the respective roles (information check). For the mistreatment type “angry insult,” we asked respondents who was angry at whom at the end of the clip. For the mistreatment type “exclusion,” we asked respondents who wanted to go on lunch with whom. Eight hundred and eighty out of 957 respondents completed at least one of the information checks correctly. A total of 590 respondents completed both information checks correctly, 171 respondents completed only the information check for mistreatment via an angry insult correctly, and 119 respondents completed only the information check for mistreatment via exclusion correctly. More specifically, for the mistreatment type of angry insult we excluded 20.48 percent of all 957 responses because the respective respondents rated the perpetrator *Christian/Christiane* as not angry, rather not angry or neither angry nor not angry at the target *Martin/Martina* or because they indicated not to know the answer (final sample *n* = 761). For the mistreatment type of exclusion, we excluded 25.91 percent of all responses as respondents answered incorrectly to the question of whether the target *Andrea/Andreas* wanted to go for lunch with the perpetrator *Daniel/Daniela* or because they indicated not to know the answer (final sample *n* = 709).

Of all 880 respondents who at least completed one information check correctly, 44.89% identified as male, 55.00% identified as female, and 0.11% identified as diverse. Age groups between 20 and 60 years were relatively evenly distributed among workforce members. 6.82% of respondents were 60 years or older, and 0.57% were younger than 20 years old. 3.64% of respondents had only compulsory education completed, 38.30% had an apprenticeship or medium-level school degree as the highest degree, 26.48% of respondents had a high school level degree, and 31.60% a university or other tertiary education degree, i.e., respondents with higher education were slightly oversampled. Of all participants, 28.86% held either a top or a middle management position. The industry with the highest share of respondents was the health sector, followed by the educational sector (approximately 10 % of respondents were from the health and the educational sector, respectively).

#### 3.1.2. Experimental design

Our 2*2*2 mixed-design includes two within-subject factors perpetrator gender and type of mistreatment, and a between-subject factor target gender. We made sure that every actor/actress who occupied a perpetrator role, also occupied at least once a target role in a clip of the same type of mistreatment. We also had at least one combination per gender configuration, where the respective actor/actress combination switched roles as targets and perpetrators. We randomized the clips and combination of actors in 32 possible ways, which led to 16 different versions for each mistreatment type. We ensured that each respondent received a female perpetrator in one mistreatment type and a male perpetrator in the other type of mistreatment (for more information on the research design please see Supplementary Table S1).

Gender effects of workplace mistreatment such as workplace bullying have mainly been studied with fictional written scenarios via manipulating the protagonists’ male or female names (e.g., Salin, 2011; Mulder et al., 2017). However, written descriptions do not fully capture the nature and the context of the mistreatment, nor do they provide any underlying insights into (gendered) attributions of blame and intent. Many mistreatment situations cannot be experimentally constructed (Sleed et al., 2002). We nevertheless found it worthwhile to study gender effects with video clips in contextualized, yet standardized conditions. Videos are richer than written vignettes and are potentially leading to more complex or conjunctive attributions of blame than a written vignette, where respondents often tend to focus on the target’s behavior and blame (Sleed et al., 2002). The advantage of “richness” in video vignettes is at the same time the major disadvantage, in particular when it comes to the study of emotionally expressive misbehavior such as anger. As such both situational noise, but also individual differences between actors and their play have to be taken into account. To make sure that none of the individual characteristics of the actors overrode possible gender effects, we employed eight professional actors (four male and four female actors) in a comparable age range (37–48 years). All actors went through casting and pre-tests where they enacted different levels and expressions of “anger” to ensure a comparable level of attractiveness and likeability, perceived competence as well as skills in enacting different types of anger levels. We also tested likeability and outcome variables in a student sample.

During the shoot in Spring 2021, all actors were dressed in similar blue or grey blouses and shirts, decent shoes, and black trousers or jeans (“business casual”). All clips were shot at the same rented office place without any other change in scenery, i.e., any noise was kept as little as possible. The first author and a professional film director directed the shooting and made sure that the form and extent of anger and harm enacted were standardized as much as possible across all actors. Two filmmakers edited and cut the final actor/actress combinations to resemble the research design (see Supplementary Table S1). Camera focus and perspective were standardized throughout, and perpetrator/target clips were cut and re-combined together, so that all scenes by one actor/actress could be combined with other actors/actresses. This also ensured standardization as the aggression and the target’s reaction displayed by each actor were consistent across all respective combinations. In addition, all 32 combinations included brief joint shoots by the respective actors to ensure that participants saw both actors together in interaction at least once.

The plots for each type of mistreatment were developed by the first author and a research assistant. They were tested and adapted in various test rounds with diverse respondents and other researchers from the workplace bullying field to make them easily understandable and brief, but containing all relevant information and ambiguous/different potential situational and individual causes of blame. Each clip lasted approximately 1.30–2 min and was preceded as well accompanied by additional written information.

### 3.2. Measures

#### 3.2.1. Mistreatment type

The first type of mistreatment is direct aggression via an angry insult. Overall, it is more expected and accepted that women display both positive and negative emotions (happiness and sadness) at work as it fits the stereotype (Zedlacher and Salin, 2021). Only anger is more expected and accepted to be displayed by men at work as it is also a sign of power (so Brescoll and Uhlmann, 2008; Sloan, 2012, see also Zedlacher and Salin, 2021). Moreover, displaying anger in organizations is subject to contextual interpretations and may involve ambiguous interpretations about the motives of the angry person, as not every anger display might be attributed to an intent to harm (see the overview on anger in organizations by Lindenbaum and Geddes, 2016).

The second type of mistreatment is about a male or female perpetrator engaging in indirect aggression in the form of exclusion (excluding the target from a work lunch). Similar to anger, ostracizing might also not have full causal clarity in the eye of the target, let alone in the eye of the observer (Sommer et al., 2001). In general striving for interpersonal relationships, community, and harmony are preferable traits associated with women (Sommer et al., 2001). Hence, women are considered to perform of social manipulation such as exclusion or gossiping more often than men, and are also considered to be more easily hurt by social manipulation than men (Salin and Hoel, 2013).

#### 3.2.2. Content of video clips

Before the introductory clip, respondents received the information that the interactions occur between different workers in the same Austrian company. Without giving details about company purpose and context, the respondents were also presented with the same possible situational attributions of blame in written form: Participants were informed that the company had suffered from delivery problems with their suppliers lately. As a result, customers were putting pressure on the perpetrator (as the one person being in direct contact with the customer). Consistent behavior and prior interactions between target and perpetrator were found to be important factors for attributions of blame both in the literature and in the pre-tests. Hence, we portrayed both the target and perpetrator as having had loose work relationships in the past without any severe conflicts. We chose all names of perpetrator and target to be easily convertible for both genders and being typical “average” Austrian names for average old workforce members.

##### 3.2.2.1. Angry insult

*Christian/e* receives a customer mail with an urgent demand for a sales list (the customer warns about changing the supplier if he does not receive the list immediately). *Christian/e* forwards the mail to his/her colleague *Martin/e* at night and asks to have the final list ready first thing in the morning. However, *Martin/a* does not send any list in the morning. When *Christian/e* enters the office and asks *Martin/a* about the list, *Martin/a* answers that s/he needs to finalize another presentation first, which makes *Christian/e* increasingly angry, and finally insults *Martin/a* (starting with “*Are you failing at everything*?!”) before leaving the room. The final camera shot is of *Martin/a* with a hurt face.

##### 3.2.2.2. Exclusion

*Daniel/a* and *Andrea/s* are colleagues and are shot during a face-to-face meeting with various (non-identifiable) customers. Respondents receive written information that customers have threatened to change suppliers for good if the deliveries would not speed up. During the meeting, *Andrea/s* asks *Daniel/a* via a short message on the mobile phone if they have lunch together. *Daniel/a* denies it by answering that s/he already has other plans. Later Andrea/s, who has lunch by him/herself in the office room, finds *Daniel/a* posting a joint lunch photo with the customers on a (non-identifiable) Social media platform. The final camera shot is of *Andrea/s* with a hurt face.

#### 3.2.2. Dependent variables

##### 3.2.2.1. Moral anger

We measured the construct of moral anger based on four items modified for third parties and already used by O’Reilly et al. (2016) and in earlier studies (e.g., Barclay et al., 2005) in both types of mistreatment. We asked participants whether the behavior shown by the perpetrator in the video made them angry via a 5-point Likert scale. Items included were “*This behavior makes me feel furious*,” “*This behavior is bad*,” “*I feel angry when I watch this behavior*,” and “*Anger builds up in me when I observe such a behavior*”. Cronbach’s Alpha for the mistreatment type of angry insult was 0.91, for the mistreatment type of exclusion Cronbach’s alpha was 0.95.

##### 3.2.2.2. Attribution of perpetrator blame

Following Shaver’s (1985) and other authors’ determinants of blame we measured perpetrator blame (intent) with 6 items on intention, foreseeability, and responsibility for harm on a 5-point Likert scale for each mistreatment type (e.g., “*Daniel posted the lunch on purpose*”; *“Christina is responsible that Martin is hurt”; “Daniela wanted to hurt Andrea”*; “*Christian could foresee the harm he would cause with his behavior”; Christiane acted with full control when she hurt Martina”; “Daniela was aware that her words will hurt Andreas”*). Cronbach’s alpha for perceived perpetrator intent was 0.81 for the angry insult, and 0.91 for exclusion, respectively. In addition, we measured the perceived internal control of harm-doing with two items (e.g., “*Daniela’s behavior was a spontaneous reflex without wanting to hurt Andrea*”; “*Christian has acted out of an impulse without consciously aiming to hurt Martin”)*. Cronbach’s alpha for perceived perpetrator control for the angry insult was 0.77, and 0.83 for exclusion.

##### 3.2.2.3. Target-blaming/coping

Target-blaming was measured with five items (5-point Likert scale) which were adapted from prior studies on workplace mistreatment with a focus on coping strategies of targets (Mulder et al., 2017). Items included were “*Andrea takes this too personally*”; *“Martin is too emotional”*; “*Andreas is to blame himself for being hurt*,” “*Martina is too sensitive”;* “*Andrea gets hurt too easily.”* Cronbach’s Alpha was 0.87 for the angry insult, and 0.91 for exclusion.

#### 3.2.3. Control variables

The following variables were taken into account as control variables in our main analyses: Observer (=respondent) gender, likeability of actors/actresses, and observers’ *Beliefs in a Just World* (Dalbert, 2000). For target-blaming, perpetrator blame (intent and control) was taken into account as covariate. For perpetrator-blaming, target-blaming was included as covariate.

##### 3.2.3.1. Observer gender

We measured gender with male, female, and diverse/other. There was one participant identifying as diverse/other. We treated this respondent as missing value in the statistical analysis as analyses were not possible with this low number.

##### 3.2.3.2. Likeability

For all actors, we first checked for the likeability and attractiveness of male actors (A, B, C, D) and female actresses (E, F, G, H) by making respondents watch a short introductory clip with a standardized business conversation in a meeting with other (non-visible) company members (i.e., before they enacted their perpetrator, respectively, target role). We used four items from the Reysen (2005) likeability scale to measure the perceived likability of actors. Items included statements such as “*Daniel is friendly*,” “*Martina is likable*,” and “*Andrea seems competent*” (ratings may range from 0 to 100). Cronbach’s Alpha for the four items was above 0.90 for all different actor names. Regarding the likeability of actors A-H, mean ranks between actors did not significantly differ with a few exceptions. The male actor A was consistently considered less likable and received more moral anger and blame than other actors did.

##### 3.2.3.3. Beliefs in a Just World

The *Beliefs in a Just World* scale (Dalbert, 2000) has been used in a variety of studies for measuring the relationship of one’s own belief that the world is fair when the respondents see more unfavorable events or situations of other people. Just-world beliefs are suggested to increase target derogation and blaming (Dhanani and LaPalme, 2019). We used the German version of the scale with a 1–7 Likert scale (Dalbert, 2000). Items included were for example “*Overall, events in my life are just*.” Cronbach’s Alpha was 0.81.

### 3.3. Statistical analyses

Linear mixed-effects modeling (LMM; West et al., 2006) was used to predict moral anger, perpetrator blame (measured via perceived intent or control of the mistreatment) as well as target-blaming. LMM was conducted in R version 4.2.3 (R Core Team, 2023) using the R package lme4 version 1.1–32 (Bates et al., 2014). Effect-coded predictors perpetrator gender (male vs. female), target gender (male vs. female), and type of mistreatment (angry insult vs. exclusion) including all two-way interaction terms and a three-way interaction term were included in the models. Effect coding method was used to obtain main effects instead of simple effects which were required to test the hypotheses of the present study. Note that mixed-effects modeling based on the effect coding method provides equivalent results as analysis of variance. The means and standard deviations of all dependent variables (moral anger, perpetrator intent, perpetrator control, and target-blaming) for each type of mistreatment and gender configuration can be found in Supplementary Table S2.

**Figure 1:**
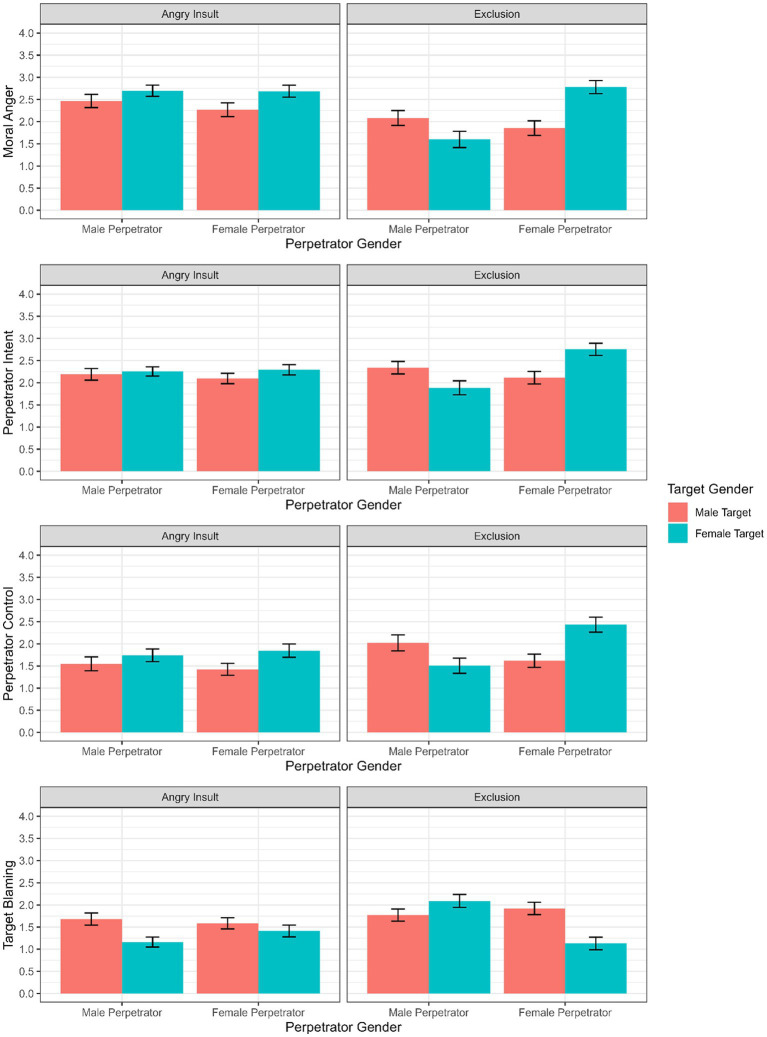
Visualization of means and standard deviations of moral anger, perpetrator intent, perpetrator control and target blaming for each perpetrator/target gender configuration without controlling for covariates.

To test our hypotheses we first ran a LMM without covariates. For a better overview, the visualization of means and standard deviations per perpetrator/target gender configuration and mistreatment type for each dependent variable without controlling for covariates can be found in Figure 1. Apart from significant main effects of perpetrator gender, we also found significant two- and three-way interactions with target gender and type of mistreatment.

**Figure 2:**
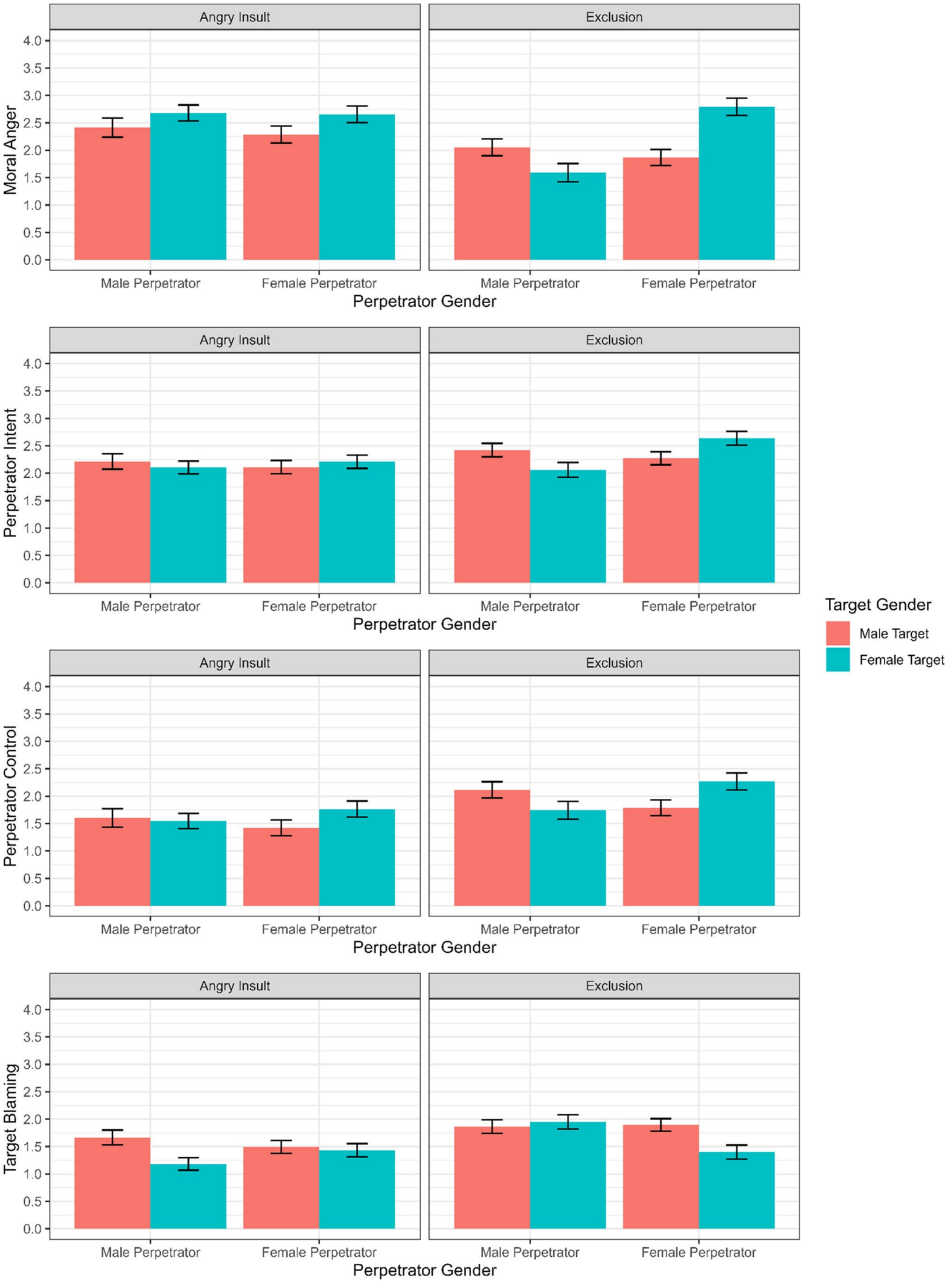
Visualization of means and standard deviations of moral anger, perpetrator intent, perpetrator control and target blaming for each perpetrator/target gender configuration while controlling for covariates.

We then added covariates to the model to test the robustness of the findings while statistically controlling for relevant factors including observer gender, likeability of actors in each mistreatment type and the respondents’ *Beliefs in a Just-World*. For perpetrator-blaming (intent and control), we controlled for target-blaming, and for target-blaming we included perpetrator-blaming (intent and control) as a covariate. The results of the model with covariates can be found in Figure 2. The findings while controlling for covariates are robust and did overall not significantly change results in the model without covariates (Figure 1). Given the equivalence of results without and with covariates, we will report the results of the models with covariates.

## 4. Results

### 4.1. Mistreatment types

Overall, both types of mistreatment both produced moderate levels of anger and blame (see Table 1). However, the outcome means significantly differed between the types (*p* < 0.001).

**Table 1:** Mean values and standard deviations of dependent variables for each mistreatment type.

Dependent variable	Angry insult		Exclusion	
	*M*	*SD*	*M*	*SD*
Moral anger	2.54	1.00	2.08	1.20
Perpetrator intent	2.21	0.81	2.28	1.02
Perpetrator controllability	1.65	1.03	1.89	1.19
Target-blaming	1.43	0.90	1.73	1.01

### 4.2. Hypotheses-testing

We first tested whether female perpetrators elicit more moral anger among observers across the types of mistreatment (1a), in particular when women mistreat female targets (1b). Table 1 lists all results of the LMM for all dependent variables. In addition, we report results of Post-Hoc tests for three-way interactions between perpetrator gender, target gender and type of mistreatment.

As Table 1 shows we found a significant main effect of perpetrator gender [Est. = 0.11, *t*(761.60) = 4.04, *p* < 0.001, $\eta_{p}^{2}$ = 0.02]. Female perpetrators (M = 2.33, 95% CI [2.22, 2.44]) triggered more observer anger than male perpetrators (M = 1.82, 95% CI [1.71, 1.94]) even when controlling for the covariates. Hence, Hypothesis 1a is confirmed. However, Table 1 shows a significant interaction between perpetrator and target gender [Est. = 0.19, *t*(764.45) = 7.06, *p* < 0.00, $\eta_{p}^{2}$ = 0.06] and a significant three-way interaction between perpetrator gender, target gender and type of mistreatment [Est. = 0.16, *t*(801.20) = 5.43, *p* < 0.001, $\eta_{p}^{2}$ = 0.04]. These results suggest that target gender, but also the mistreatment type needs to be taken into account when assessing the impact of perpetrator gender on an observer’s moral anger. Post-hoc tests give more insights into the direction of the moral anger: For female perpetrators, the amount of moral anger they triggered among observers depended on the target gender and the type of mistreatment: For the angry insult, a female perpetrator of a female target did not receive more blame than a male perpetrator of the female target [*t*(1413) = 0.23, *p* = 1.000]. However, for exclusion, a female perpetrator (*M* = 2.79, 95% CI [2.63, 2.95]) of a female target received much more moral anger than a male perpetrator of the woman (*M* = 1.59, 95% CI [1.42, 1.76]) [*t*(1423) = −10.29, *p* < 0.001]. Hence, hypothesis 1b is only partly confirmed (exclusion). In addition, we tested the effect of a male target on moral anger against the perpetrator. When the target was male the perpetrator gender had no significant impact on the observer’s moral anger neither for the angry insult [*t*(1425) = 1.08, *p* = 0.961] nor for the exclusion [*t*(1417) = 1.71, *p* = 0.680].

In the next step, we tested whether perpetrator-blaming (perceived intent and control) is stronger against female perpetrators than against male perpetrators (Hypothesis 2a), in particular when the mistreatment is performed against female targets (Hypothesis 2b).

As Table 2 shows, we found a significant main effect of perpetrator gender on attributions of intent [Est. = 0.05, *t*(774.27) = 2.58, *p* = 0.010, $\eta_{p}^{2}$ = 0.00], but not on perceived perpetrator control of the mistreatment [Est. = 0.03, *t*(780.43) = 1.13, *p* = 0.257, $\eta_{p}^{2}$ = 0.00]. Hence, hypothesis 2a is confirmed for perpetrator intent, but rejected for perpetrator control. We also found significant interactions between perpetrator and target gender for both attributions of intent [Est. = 0.12, *t*(774.95) = 5.65, *p* < 0.001, $\eta_{p}^{2}$ = 0.04] and perceived control of the mistreatment [Est. = 0.16, *t*(781.65) = 6.00, *p* < 0.001, $\eta_{p}^{2}$ = 0.04], and significant three-way interactions between perpetrator gender, target gender and type of mistreatment for the attribution of intent [Est. = 0.06, *t*(841.52) = 2.67, *p* = 0.008, $\eta_{p}^{2}$ = 0.00], and for perceived controllability of the mistreatment [Est. = 0.06, *t*(822.18) = 2.00, *p* = 0.046, $\eta_{p}^{2}$ = 0.00]. Post-hoc tests revealed that for the angry insult, female and male perpetrators of female targets did not differ with regard to attributed intent [*t*(1398) = −1.24, *p* = 0.919] nor control [*t*(1417) = −2.12, *p* = 0.402]. However for the mistreatment type exclusion, the gender of the perpetrator and the target affected perceptions of perpetrator intent significantly: Female perpetrators (*M* = 2.64, 95% CI [2.51, 2.77]) of female targets were attributed much more intent than male perpetrators (*M* = 2.06, 95% CI [1.93, 2.19]) of female targets [*t*(1414) = −6.05, *p* < 0.001], and also much more control (*M* = 2.27, 95% CI [2.11, 2.42] vs. *M* = 1.74, 95% CI [1.58, 1.90]) [*t*(1420) = −4.56, *p* < 0.001] for the exclusion (Table 2). Hence, Hypothesis 2b is confirmed for the mistreatment type of exclusion, but not for mistreatment via an angry insult.

**Table 2:** Linear mixed effects modeling for moral anger against perpetrator.

Moral anger	Estimate	SE	df	*t-* value	*p*-value
(Intercept)	2.21	0.08	803.17	28.99	<0.001
Mistreatment type	−0.22	0.03	772.95	−7.98	<0.001
Perpetrator gender	0.11	0.03	761.60	4.04	<0.001
Target gender	0.14	0.03	801.84	4.64	<0.001
Observer gender	0.12	0.06	806.44	2.09	0.0369
Likeability of actors (angry insult)	−0.01	0.00	735.94	−4.07	<0.001
Likeability of actors (exclusion)	0.00	0.00	746.65	1.11	0.266
Beliefs in a just world	0.01	0.04	791.91	0.38	0.702
Mistreatment type * perpetrator gender	0.15	0.03	802.54	4.93	<0.001
Mistreatment type * target gender	−0.02	0.03	764.02	−0.83	0.406
Perpetrator * target gender	0.19	0.03	764.45	7.05	<0.001
Mistreatment type * perpetrator * target gender	0.16	0.03	801.20	5.43	<0.001

In alignment with moral anger, when the target was male the observers attributed similar levels of intent and control to male and female perpetrators.

Finally, we investigated the impact of perpetrator gender on gender biases in target-blaming (hypothesis set 3). We hypothesized that targets of female perpetrators would receive less blame than targets of male perpetrators (Hypothesis 3a), in particular when the target identified as female rather than male (Hypothesis 3b). Covariates were observer gender, likeability of actors for each type of mistreatment, *Beliefs in a just World* as well as perpetrator-blaming. As Table 3 shows, there was a significant main effect of perpetrator gender on target-blaming, i.e., targets of female perpetrators were blamed less than targets of male perpetrators [Est. = −0.06, *t*(748.89) = −2.82, *p* = 0.005, $\eta_{p}^{2}$ = 0.01]. Hence, hypothesis 3a is confirmed. While we did not find a significant two-way interaction between perpetrator and target gender, we found a significant three-way interaction between perpetrator gender, target gender and type of mistreatment [Est. = −0.13, *t*(824.66) = −5.19, *p* < 0.001, $\eta_{p}^{2}$ = 0.03]. Hence, in alignment with perpetrator-blaming, for target-blaming all three manipulated variables have to be taken into account in the study of gender biases. Post-hoc tests revealed no significant effect of perpetrator gender on the blaming of the female target for the angry insult [*t*(1372) = −3.00, *p* = 0.055]. However, a female target of a female perpetrator (*M* = 1.40, 95% CI [1.27, 1.53]) was blamed significantly less for being excluded than a female target of a male perpetrator (*M* = 1.95, 95% CI [1.82, 2.08]) [*t*(1406) = 5.92, *p* < 0.001].

**Table 3:** Linear mixed effects modeling for perceived perpetrator intent and controllability.

	Estimate	SE	df	*t*-value	*p*-value
Perpetrator-blaming (intent)					
(Intercept)	2.74	0.07	989.29	38.76	0.000
Mistreatment type	0.09	0.02	799.27	4.42	0.000
Perpetrator gender	0.05	0.02	774.27	2.58	0.010
Target gender	0.00	0.02	835.98	−0.03	0.976
Observer gender	−0.13	0.05	834.46	−2.66	0.008
Likeability of actors (angry insult)	−0.01	0.00	745.79	−3.42	0.001
Likeability of actors (exclusion)	0.00	0.00	748.37	1.04	0.297
Beliefs in a just world	0.09	0.03	829.35	2.86	0.004
Target-blaming	−0.36	0.03	1404.94	−14.16	0.000
Mistreatment type * perpetrator gender	0.05	0.02	828.12	2.20	0.028
Mistreatment type * target gender	0.00	0.02	767.64	0.09	0.925
Perpetrator * target gender	0.12	0.02	774.95	5.65	0.000
Mistreatment type * perpetrator gender * target gender	0.06	0.02	841.52	2.67	0.008
Perpetrator-blaming (control)					
(Intercept)	2.54	0.08	963.98	30.54	0.000
Mistreatment type	0.20	0.03	805.32	7.36	0.000
Perpetrator gender	0.03	0.03	780.43	1.13	0.257
Target gender	0.05	0.03	816.79	1.75	0.080
Observer gender	−0.15	0.06	817.32	−2.73	0.006
Likeability (angry insult)	0.00	0.00	750.35	−1.05	0.292
Likeability (exclusion)	0.00	0.00	753.21	0.16	0.870
Beliefs in a just world	0.00	0.04	810.74	0.09	0.931
Target-blaming	−0.43	0.03	1382.96	−14.32	0.000
Mistreatment type * perpetrator gender	0.02	0.03	809.26	0.72	0.473
Mistreatment type * target gender	−0.02	0.03	774.43	−0.88	0.382
Perpetrator gender * target gender	0.16	0.03	781.65	6.00	0.000
Mistreatment type * perpetrator gender* target gender	0.06	0.03	822.18	1.99	0.046

Regarding our covariates included in the model, we found a main effect of observer gender for moral anger, perpetrator control as well as target-blaming. Table 1 shows that female observers tended to have more deontic responses in the form of moral anger [Est. = 0.12, *t*(806.44) = 2.09, *p* = 0.037, $\eta_{p}^{2}$ = 0.00]. However, they seemed less inclined to engage in (cognitive) perpetrator- and target-blaming than men (Tables 2, 3). The likeability of actors significantly affected moral anger and perpetrator intent for the first type of mistreatment (Tables 1, 2). In other words, the higher the likeability of the actors, the less the moral anger against the perpetrator and the less the perceived intent for insulting the co-worker.

*Beliefs in a Just World* were positively correlated with perpetrator intent and target-blaming (see Tables 3, 4). In line with prior literature, the more respondents endorsed just-world beliefs, the more likely they were to blame the target (see also Dhanani and LaPalme, 2019).

**Table 4:** Linear mixed effects modeling for target-blaming.

Target-blaming	Estimate	SE	df	*t*-value	*p*-value
(Intercept)	2.21	0.08	1189.57	26.74	0.000
Mistreatment type	0.17	0.02	763.30	8.31	0.000
Perpetrator gender	−0.06	0.02	748.89	−2.82	0.005
Target gender	−0.12	0.02	821.36	−4.94	0.000
Observer gender	−0.26	0.05	815.27	−5.43	0.000
Likeability (angry insult)	0.00	0.00	726.63	1.94	0.053
Likeability (exclusion)	0.00	0.00	724.06	−0.48	0.634
Beliefs in a just world	0.20	0.03	812.42	6.55	0.000
Perpetrator intent	−0.21	0.03	1402.00	−6.54	0.000
Perpetrator controllability	−0.18	0.03	1408.58	−6.66	0.000
Mistreatment type * perpetrator gender	−0.08	0.02	821.41	−3.12	0.002
Mistreatment type * target gender	0.02	0.02	740.67	0.86	0.390
Mistreatment type * target gender	−0.02	0.02	768.98	−1.00	0.319
Mistreatment type * perpetrator * target gender	−0.13	0.02	824.66	−5.19	0.000

Finally, we find perpetrator- and target-blaming highly negatively correlated, pointing to uni-directional rather than conjunctive blame patterns in this experiment.

### 4.3. Exploratory analysis regarding observer gender

We ran additional exploratory analyses regarding the effect of observer gender on outcome variables and the interplay with perpetrator gender, target gender, and mistreatment type. Results showed no statistically significant two-, three-, or four-way interactions involving observer gender on any of the outcome variables. Hence, while there was a main effect of observer gender on moral anger and blame, this observer gender bias was not affected by the gender of the perpetrator, the gender of the target, or by the type of the mistreatment.

## 5. Discussion

The main goal of this empirical study was to explore the impact of perpetrator gender on observers’ blame attributions for workplace mistreatment among co-workers. Our main proposition founded in social role theory (Eagly and Wood, 2012) and attribution theory (Kelley and Michela, 1980) was that female perpetrators would elicit more moral anger and attributions of intent and control among observers than male perpetrators, irrespective of whether the aggression was via an angry insult or exclusion.

Overall both types of mistreatment triggered “moderate” levels of moral anger and blame against the perpetrator as expected. However, the first type of mistreatment (insult) produced more moral anger, and the second mistreatment type (exclusion) produced higher means of perpetrator intent. This suggests that moral anger as a quick deontic response versus the cognitive evaluation of intent and controllability are distinct processes (see also Haidt, 2001).

We found a main effect of perpetrator gender for moral anger and intent, though it should be noted that the effect sizes are rather small. Importantly, the effect of perpetrator gender is dependent on both the gender of the target and on the type of mistreatment, with larger effect sizes for moral anger than for perpetrator-blaming. In other words, female perpetrators were judged more harshly than men when the target was female and the mistreatment type exclusion. The overall lack of perpetrator gender effects for the mistreatment type angry insult is surprising since the expression of anger may not only be less expected/acceptable from women (Brescoll and Uhlmann, 2008), but also be perceived as more painful for a female target due to socialization. However, if an insult is considered as an angry *outburst*, the perceived lack of control of an actor’s outburst or impulse (Malle et al., 2014) may have overridden possible gender effects. In contrast, for the mistreatment type exclusion not only was moral anger (and blame) stronger against women, but even target-blaming seemed to follow a gendered pattern: A female target received significantly less blame when the perpetrator was female rather than male. One explanation for these findings is the context and content of the mistreatment itself: Social exclusion by and of women has been found common, yet hurtful (Salin and Hoel, 2013). Exclusion of a female peer might lead to more negative perceptions than rejecting lunch with male peers due to the violation of communal and “inclusive” behavior and solidarity among women. Moreover, the “notorious” problematization of female/female harassment in the popular discourse and the alleged lack of solidarity between women may have affected observers’ evaluations of mistreatment via exclusion, and corroborates earlier experimental findings (Sheppard and Aquino, 2013). Also, at least for this type of mistreatment we find Salin’s (2011, 2021) claim corroborated that a female target of a male perpetrator is a configuration that seems to evoke more target-blaming than other configurations. This might be owed to the attribution of hypersensitivity or other stereotypical traits to women when excluded by “thoughtless” men (Zedlacher and Salin, 2021). In contrast to female targets, male targets of interpersonal mistreatment at work did not trigger such gender biases. Female targets of male perpetrators may appear less credible than male targets when they get hurt. Just as perpetrator-blaming, the construction of an “ideal” victim is a social and ideological process (Daly, 2014; Christie, 2018) and requires further investigation for (subtle) workplace mistreatment and gender bias. Our study findings suggest extending the study of victim/target-blaming to stereotypes about wrong coping and continuation responsibility (Mulder et al., 2017) rather than a sole focus on dispositional attributions and causes of mistreatment in the target’s “personality.”

Regarding our control variables, we found a main effect of observer gender on outcome variables, yet the effect size was small. Women tended to engage less in blaming perpetrators and targets than men, i.e., while moral anger might be high, women might still refrain from attributing intent to the parties involved. It is known from prior experimental studies on mistreatment that women tend to engage less in target-blaming than men and often attribute situational/external causes (e.g., Salin, 2011). With regard to perpetrator-blaming, further (qualitative) research can shed light on whether female observers tend to engage in situational attributions and explanations (e.g., customer pressure) more than men which may lower women’s attributions of intent and controllability of “aggressive” behavior. It is noteworthy, that no significant interactions between observer gender and perpetrator gender, target gender and type of mistreatment were found in our study. This is different from prior studies in the mistreatment context which suggest same sex-identification between observer and target or perpetrator (see Salin, 2011; Grubb and Turner, 2012). It is also important to note, that other covariates such as the likeability of actors or the observer’s *Beliefs in a Just World* influenced observer perceptions of the target and the perpetrator. As expected target-blaming was related to high just-world beliefs. Future studies can explore why perceptions of perpetrator intent relate to *Beliefs in a Just World*, but perceived controllability does not.

We have argued before that in ambiguous workplace mistreatment, the roles of “perpetrators” and “targets” are dynamic and complex rather than fixed. In our study, we find a clear negative relationship between perpetrator and target-blaming, i.e., respondents who attributed much blame to the perpetrator engaged less in target-blaming and vice versa. Nevertheless, our patterns in perpetrator- and target-blaming imply that conjunctive blaming (blaming one dominant actor, and in addition/conjunction blaming others; Wilkerson and Meyer, 2019) was prevalent in the study, i.e., no actor was fully excused from the wrongdoing. This pattern of shifting blame and/or conjunctive attributions of blame might even become stronger if mistreatment and interactions last longer than a video clip and actors provide more informational cues for their behavior (Zedlacher and Snowden, 2023).

This study also provides important evidence and a new focus in organizational anti-bias trainings concerning female/female harassment stereotypes and victim/target-blaming. Trivializing conflicts between women as cat fights or immediately blaming the female target of an “assertive” male perpetrator may lead to a lack of or inadequate intervention by third parties (e.g., Mawdsley and Thirlwall, 2019; Zedlacher and Snowden, 2023). According to Lindenbaum and Geddes (2016), moral anger is a primary appraisal of a moral standard violation that affects others more than oneself and triggers corrective behavior (irrespective of personal risk). Our study findings show that in particular moral anger as a quick deontic response (Haidt, 2001) and evaluations of targets’ reactions are likely to produce unconscious gender bias, but might be weakened through post-hoc reasoning. In addition, trainings should also create awareness for male health and tackle potential barriers for men to report victimization due to social role pressures.

It is important to note the limitations of this study. In general, each of the two types of mistreatment depicted a very specific context and form of misbehavior at work. Other types of potentially ambiguous negative workplace interactions are required to gain more insights into gender dynamics. While both types of mistreatment entailed the same situational pressure (customers threatening to change to other suppliers), the distress caused by this situational factor in the angry insult episode might have been higher due to the personalized email from the customer to the perpetrator, whereas in the exclusion clip the customer pressure was only explained in written form. Moreover, while we received small to medium effect sizes for moral anger and target-blaming, effect sizes for three-way interactions in perpetrator-blaming were very small. Finally, contextualizing workplace mistreatment in video experiments may make the setting and context more realistic, but it might come at the expense of the generalizability of findings. Regarding the effect of actor characteristics on findings, we found in particular the male actor A (the least likable actor) to elicit a significant higher level of perpetrator-blaming in the first type of mistreatment (angry insult). However, overall, we are positive that the research design and the multitude of actors and standardized shots and combinations have ruled out individual characteristics and likeability differences.

## 6. Conclusion

Assigning blame to a “perpetrator” or a “target” for mistreatment at work is often difficult from an observer standpoint, and gender biases in attributions may manifest subtly. This study’s findings indicate that both perpetrator and target gender affect moral anger and create attributional biases, yet the type of mistreatment is equally decisive on whether and how gender biases occur. Our standardized video experiments with eight professional actors found that women were judged and blamed equally to men for insulting their co-workers. However, women were blamed more then men for excluding their co-workers from lunch when the co-worker was female, corroborating persistent stereotypes about women harassing other women. While this study focused on exploring the effect of perpetrator gender, the findings highlight the salient role of the target gender for observer reactions and perpetrator-blaming. While experimental combinations with male targets had no effect on attributional biases, female targets received less blame for their coping when the perpetrator was female, but were seen as (too) sensitive when the perpetrator was male. As such, this study offers important avenues for future research and practice. More studies on gender biases in target-blaming that arise from a target’s inadequate reactions to the mistreatment are necessary. Organizations need to train their members regarding attributional biases and stereotypes for specific perpetrator/target gender combinations.

* We purposefully use the term “target” for receivers of ambiguous mistreatment as it does not imply (claiming) victim status (*cf.* Christie, 2018; for a further discussion of targets vs. victims please see Nielsen et al., 2020). We use the term “victim” or “victim-blaming” when referring to studies where the respective terms were used.

## Data availability statement

The raw data supporting the conclusions of this article will be made available by the authors, without undue reservation.

## Ethics statement

The studies involving human participants were reviewed and approved by the Institutional Review Board (Webster University). The patients/participants provided their written informed consent to participate in this study.

## Author contributions

EZ: planning and execution of the study, theory and hypotheses, interpreting results, and writing—original draft preparation. TY: planning of the study, hypotheses, analyzing data, writing-up results. All authors contributed to the article and approved the submitted version.

## Funding

This video experiments have been funded by the *Projektfonds Arbeit 4.0* grant from the *Austrian Chamber of Labour* (section Lower Austria) and by the *Webster Faculty Research Grant* 2020 and 2021.

## Conflict of interest

The authors declare that the research was conducted in the absence of any commercial or financial relationships that could be construed as a potential conflict of interest.

## Publisher’s note

All claims expressed in this article are solely those of the authors and do not necessarily represent those of their affiliated organizations, or those of the publisher, the editors and the reviewers. Any product that may be evaluated in this article, or claim that may be made by its manufacturer, is not guaranteed or endorsed by the publisher.
