# Supplementary material for: Gender biases in attributions of blame for workplace mistreatment: a video experiment on the effect of perpetrator and target gender
Source: Front Psychol. 2023 Jun 30;14:1161735. doi: 10.3389/fpsyg.2023.1161735 (PMC10349265; doi:10.3389/fpsyg.2023.1161735)
Supplement: Supplementary file 1 [file Data_Sheet_1.pdf]

Supplementary Table 1

*Research design: 2*2*2 mixed subjects experiments including all actor/actress combinations*

Angry Insult		Exclusion		Angry Insult		Exclusion		
Perpe-trator Gender	Target Gender	Perpe-trator Gender	Target Gender	Perpe-trator Code	Target Code	Perpe-trator Code	Target Code	Combination
Male	Male	Female	Male	A	B	E	D	1
				B	A	F	C	2
				C	D	G	A	3
				D	C	H	B	4
Female	Male	Male	Male	F	B	A	D	5
				E	A	B	C	6
				G	D	C	B	7
				H	C	D	A	8
Male	Female	Female	Female	A	F	E	G	9
				B	E	F	H	10
				C	H	G	E	11
				D	G	H	F	12
Female	Female	Male	Female	E	H	A	G	13
				F	G	B	H	14
				G	F	C	E	15
				H	E	D	F	16

Legend: A-D signify male actors, F-H females

Supplementary Table 2

Mean values and standard deviations of dependent variables for all perpetrator/target configurations

Mistreatment Type		Angry Insult						Exclusion					
Target Gender		Male Target			Female Target			Male Target			Female Target		
Dependent Variables	Perpetrator Gender	n	M	SD	n	M	SD	n	M	SD	n	M	SD
Moral Anger	Male Perpetrator	145	2.47	0.91	221	2.70	0.96	180	2.08	1.14	156	1.60	1.16
	Female Perpetrator	192	2.27	1.09	200	2.69	0.97	198	1.85	1.18	171	2.78	0.99
Perpetrator Intent	Male Perpetrator	144	2.19	0.79	221	2.25	0.78	179	2.34	0.96	156	1.89	1.00
	Female Perpetrator	191	2.10	0.82	197	2.29	0.83	199	2.11	1.01	171	2.75	0.92
Perpetrator Control-lability	Male Perpetrator	144	1.55	0.96	221	1.74	1.08	179	2.02	1.22	156	1.51	1.09
	Female Perpetrator	191	1.42	0.93	197	1.85	1.06	199	1.62	1.08	171	2.43	1.13
Target Blaming	Male Perpetrator	141	1.68	0.83	219	1.16	0.85	178	1.77	0.94	156	2.09	0.92
	Female Perpetrator	190	1.58	0.87	197	1.41	0.94	197	1.92	0.99	172	1.13	0.94
